# Carvedilol increases seizure resistance in a mouse model of *SCN8A*-derived epilepsy

**DOI:** 10.3389/fphar.2024.1397225

**Published:** 2024-06-04

**Authors:** Jennifer C. Wong, Andrew Escayg

**Affiliations:** Department of Human Genetics, Emory University, Atlanta, GA, United States

**Keywords:** *SCN8A*, epilepsy, carvedilol, amitriptyline, seizure susceptibility

## Abstract

Patients with mutations that alter the function of the sodium channel *SCN8A* present with a range of clinical features, including mild to severe seizures, developmental delay, intellectual disability, autism, feeding dysfunction, motor impairment, and hypotonia. In an effort to identify compounds that could be potentially beneficial in *SCN8A-*associated epilepsy, Atkin et al. conducted an *in vitro* screen which resulted in the identification of 90 compounds that effectively reduced sodium influx into the cells expressing the human *SCN8A* R1872Q mutation. The top compounds that emerged from this screen included amitriptyline, carvedilol, and nilvadipine. In the current study, we evaluated the ability of these three compounds to increase resistance to 6 Hz or pentylenetetrazole (PTZ)-induced seizures in wild-type CF1 mice and in a mouse line expressing the human *SCN8A* R1620L mutation. We also evaluated the effects of fenfluramine administration, which was recently associated with a 60%–90% decrease in seizure frequency in three patients with *SCN8A*-associated epilepsy. While amitriptyline, carvedilol, and fenfluramine provided robust protection against induced seizures in CF1 mice, only carvedilol was able to significantly increase resistance to 6 Hz- and PTZ-induced seizures in RL/+ mutants. These results provide support for further evaluation of carvedilol as a potential treatment for patients with *SCN8A* mutations.

## Introduction

The first *SCN8A-*associated epilepsy mutation was identified in 2012 (Veeramah et al., 2012), and since then, over 250 *SCN8A* mutations have been reported (Johannesen et al., 2022). *SCN8A* encodes the voltage-gated sodium channel (VGSC) Na_v_1.6 which is localized to the axon initial segment and the nodes of Ranvier (Caldwell et al., 2000; Boiko et al., 2001; Boiko et al., 2003) where it plays an important role in regulating neuronal excitability (Caldwell et al., 2000; Boiko et al., 2001; Boiko et al., 2003). Patients with *SCN8A* mutations present with a wide range of clinical features, including mild to severe seizures, developmental delay, intellectual disability, autism, feeding dysfunction, motor impairment, and hypotonia (Johannesen et al., 2022; Andrews et al., 2023). Thus, it is possible that multiple treatment strategies may be necessary to effectively address the range of clinical phenotypes. Gain-of-function *SCN8A* mutations have been shown to increase persistent and resurgent sodium currents *in vitro* and *in vivo* (Lopez-Santiago et al., 2017; Liu et al., 2019; Wengert et al., 2019), and drugs that reduce these sodium currents can mitigate neuronal hyperexcitability (Wengert et al., 2019). While randomized clinical trials for *SCN8A*-associated disease have not yet been completed, high doses of sodium channel blockers are efficacious in some patients. In a study by Gardella and others, the sodium channel blockers oxcarbazepine, carbamazepine, and phenytoin, or benzodiazepines provided the best seizure control in a cohort of 22 patients with *SCN8A* mutations (Gardella et al., 2018).

In an effort to find additional compounds that could be beneficial in *SCN8A-*associated epilepsy, Atkin et al., conducted an *in vitro* screen of 1,320 pharmaceutical compounds to identify those that reduce sodium influx into HEK293 cells transfected with constructs expressing either wild-type *SCN8A* or the *SCN8A* R1872Q mutation. From this screen, 90 compounds displayed inhibition at a level greater than twice the standard deviation (≥63% inhibition). Amitriptyline, carvedilol, and nilvadipine emerged as lead compounds, and additional electrophysiological analyses showed that these three drugs inhibited sodium currents at levels that were comparable to carbamazepine, an established anti-seizure drug (Atkin et al., 2018). In addition, the serotonergic drug fenfluramine is gaining interest as a possible treatment for *SCN8A*-associated epilepsy. Previously used in the treatment of depression and obesity, fenfluramine recently received Food and Drug Administration (FDA) approval for the treatment of Dravet syndrome, Lennox-Gastaut syndrome, and Tuberous Sclerosis Complex. In a recent case report, fenfluramine was found to decrease seizure frequency by 60%–90% in three patients with *SCN8A*-associated epilepsy (Aledo-Serrano et al., 2022).

In the current study, we evaluated the ability of amitriptyline, carvedilol, nilvadipine, and fenfluramine to increase resistance to induced seizures in CF1 wild-type (WT) mice and in mice expressing the human *SCN8A* R1620L mutation which was identified in a patient with behavioral seizures, ADHD, autism, and social behavior challenges (Rossi et al., 2017). We previously reported that heterozygous mutants (RL/+) exhibit increased susceptibility to induced seizures, infrequent spontaneous seizures, and several behavioral deficits (Wong et al., 2021a). We selected the RL/+ mutants for this *in vivo* drug screen as these mutants do not exhibit the high rate of premature death observed in other *SCN8A* epilepsy mouse models (Wagnon et al., 2015; Bunton-Stasyshyn et al., 2019). Furthermore, the R1620L mutation is associated with both gain-of-function and loss-of-function properties (Liu et al., 2019; Wong et al., 2021a); thus, we speculated that the RL/+ mutants would provide the opportunity to identify drugs that could be broadly therapeutic. In the current study, we found that amitriptyline, carvedilol, and fenfluramine increased resistance against induced seizures in WT mice. However, only carvedilol was able to provide significant protection against 6 Hz- and pentylenetetrazole-induced seizures in the RL/+ mutants.

## Materials and methods

### Animals

Two-month-old wild-type CF1 males (Stock No. 023, Charles River) were used to generate the dose-response curves and for the 6 Hz and pentylenetetrazole (PTZ) seizure induction experiments. Heterozygous *Scn8a* R1620L (RL/+) mutants and WT littermates (2–4 months) at the N10 generation were used for 6 Hz and PTZ seizure induction. RL/+ mutants and WT littermates were genotyped as previously described (Wong et al., 2021a) and maintained on a C57BL/6J background (Stock No. 000664, Jackson Laboratories). Mice were housed on a 12H light-dark cycle and food and water were provided *ad libitum*. All experiments were conducted in accordance with the guidelines of the Emory University Institutional Animal Care and Use Committee and the Animal Research: Reporting of *In Vivo* Experiments (ARRIVE) guidelines.

### 6 Hz seizure induction

6 Hz seizures were induced as previously described (Wong et al., 2016; Lamar et al., 2017; Shapiro et al., 2019; Wong et al., 2019; Inglis et al., 2020; Wong et al., 2021a; Wong et al., 2021b; Shapiro et al., 2022; Shiu et al., 2022). A topical anesthetic (proparacaine hydrochloride) was administered to the cornea of the mice prior to corneal stimulation (6 Hz, 0.2 ms pulse width, 3 s) using a constant current device (ECT unit, 57800; Ugo Basile, Comerio, Italy). Following corneal stimulation, behavioral seizure responses were scored using a modified Racine scale (RS): RS0, no behavioral seizure response, RS1, immobile ≥3 s, RS2, forelimb clonus, head bobbing, paw waving; and RS3, generalized tonic-clonic seizure (GTCS) with loss of posture. CF1 mice were tested at current intensities of 22 or 44 mA, and RL/+ mutants and WT littermates were tested at 16 mA.

### Pentylenetetrazole seizure induction

Pentylenetetrazole (PTZ) seizure induction was performed as previously described (Wong et al., 2016; Shapiro et al., 2019; Wong et al., 2019; Wong et al., 2021a; Wong et al., 2021b; Shapiro et al., 2022). PTZ was dissolved in 0.9% saline and administered subcutaneously to CF1 mice (85 mg/kg) and RL/+ mutants and WT littermates (100 mg/kg). Mice were observed for 30 min, and the latencies to the first myoclonic jerk (MJ) and GTCS were recorded.

### Pharmaceutical compounds

Amitriptyline hydrochloride (Fisher Scientific) and fenfluramine hydrochloride (Millipore Sigma) were dissolved in 0.9% saline. Carvedilol (VWR) and nilvadipine (VWR) were dissolved in 30% DMSO and 0.9% saline. All pharmaceutical compounds were administered intraperitoneally.

### Statistical analyses

 For dose-response curves, a Kruskal-Wallis test followed by Dunn’s multiple comparisons was used to compare the effect of different doses of each drug on Racine scores following 6 Hz seizure induction in CF1 mice. A Mann-Whitney test was used to compare vehicle and treatment following 6 Hz seizure induction in CF1 mice. A Friedman’s test was used to compare the effect of treatment on 6 Hz seizures in the RL/+ mutants and WT littermates. A log-rank Mantel-Cox test was used to compare the effect of vehicle and treatment on PTZ-induced seizures. Data are presented as mean ± SEM with *p* ≤ 0.05.

## Results

### Amitriptyline and carvedilol protect against induced seizures in CF1 mice

We selected amitriptyline (AMI), carvedilol (CVD), and nilvadipine for testing in the *Scn8a* ^R1620L/+^ (RL/+) mouse line based on a previous *in vitro* screen that suggested these drugs might be efficacious in *SCN8A*-derived epilepsy due to their ability to inhibit sodium influx (Atkin et al., 2018). Fenfluramine (FF) was selected because it is efficacious in patients with Dravet syndrome and Lennox-Gastaut syndrome (Ceulemans et al., 2012; Ceulemans et al., 2016; Knupp et al., 2022; Knupp et al., 2023), and was recently reported to reduce seizure frequency in three patients with *SCN8A*-derived epilepsy (Aledo-Serrano et al., 2022).

**FIGURE 1:**
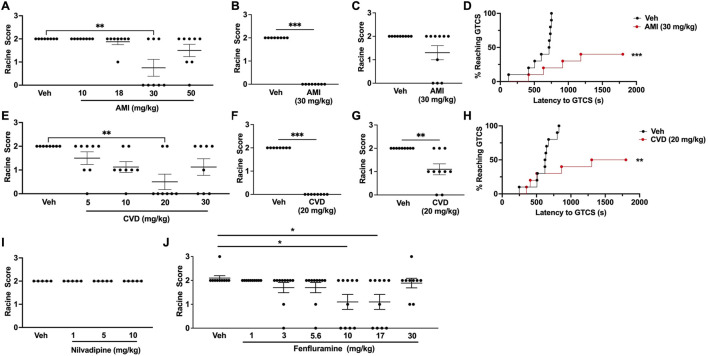
Generation of dose-response curves in CF1 wild-type mice. **(A)** Treatment with 30 mg/kg amitriptyline (AMI) was able to significantly increase resistance against 6 Hz-induced seizures in CF1 wild-type (WT) mice. *N* = 8/group. **(B)** In a separate cohort of mice, we confirmed that 30 mg/kg AMI was able to protect against 6 Hz seizures (22 mA). *N* = 8/group. **(C)** When tested at twice the convulsive current (2xCC, 44 mA), 30 mg/kg AMI did not protect against 6 Hz seizures. *N* = 9/group. **(D)** 30 mg/kg AMI was able to increase the latency to the first generalized tonic-clonic seizure (GTCS) following administration of pentylenetetrazole (PTZ). Six of 10 AMI-treated mice were completely protected against PTZ-induced seizures. *N* = 10/group. **(E)** Carvedilol (CVD, 20 mg/kg) was able to significantly increase resistance against 6 Hz seizures. *N* = 8/group. **(F)** We confirmed 20 mg/kg CVD protects against 6 Hz-induced seizures in a separate cohort of mice (22 mA). *N* = 8/group. **(G)** At 2xCC, 20 mg/kg CVD also significantly increased resistance against 6 Hz seizures. *N* = 9–10/group. **(H)** CVD (20 mg/kg) was also able to increase the latency to the first GTCS following PTZ administration. Of the CVD-treated mice, 50% did not exhibit a GTCS during the 30-min observation period. *N* = 10/group. **(I)** Nilvadipine did not increase resistance against 6 Hz-induced seizures at the doses tested. *N* = 5/group. **(J)** We found that 10 and 17 mg/kg fenfluramine was able to protect against 6 Hz seizures. *N* = 9–10/group. *p ≤ 0.05, **p < 0.01, ***p < 0.001.

Table 1 provides a summary of all of the results. For each drug, we first generated a ¼ log dose-response curve based on the ability to increase resistance to 6 Hz seizures in CF1 mice. The range of drug doses tested was based on previous studies in mouse models of other neurological disorders (Parra et al., 2002; El-Kharashi and Abd El Samad, 2011; Goel et al., 2011; Goel, 2013; Vanelderen et al., 2013; Morin et al., 2018; Morin et al., 2020). We found that significant protection against 6 Hz seizures was achieved at a dose of 30 mg/kg AMI; five of 8 CF1 mice treated with this dose did not exhibit a seizure (RS0; Figure 1A). In a separate cohort of CF1 mice, we found that 30 mg/kg AMI prevented 6 Hz-induced seizures in all treated mice, confirming effective protection (Figure 1B). We next tested whether 30 mg/kg AMI could also protect against 6 Hz seizures induced at twice the convulsive current (2xCC, 44 mA), which is a current intensity previously shown in CF1 mice to be predictive of drugs that might be efficacious in refractory epilepsies (Barton et al., 2001). However, while 33% (3/9) of AMI-treated mice did not exhibit a behavioral seizure at 2xCC (RS0 score), this was not statistically different from the response of vehicle-treated CF1 mice (Figure 1C). Finally, we found that AMI (30 mg/kg) administration also resulted in a robust increase in the latency to the first GTCS following PTZ administration in the CF1 mice (Figure 1D). Notably, six of 10 (60%) of AMI-treated CF1 mice did not have a GTCS during the 30-min observation, while all vehicle-treated CF1 mice exhibited a GTCS.

**TABLE 1:** Summary of *in vivo* drug screen in CF1 mice and *Scn8a* ^R1620L/+^ (RL/+) mutants.

Mouse	Drug	Dose (mg/kg)	Approximate human dose (mg/kg)	Seizure induction paradigm	Results	Significance
CF1	Amitriptyline	10–50	0.8–4.1	6 Hz (22 mA)	30 mg/kg AMI increased seizure resistance	*p* < 0.01
CF1	Amitriptyline	30	2.4	6 Hz (22 mA)	↑ seizure resistance	*p* < 0.001
				6 Hz (44 mA)	No statistically significant difference	NS
				PTZ (85 mg/kg)	↑ latency to first GTCS	*p* < 0.001
CF1	Carvedilol	5–30	0.1–2.4	6 Hz (22 mA)	20 mg/kg CVD increased seizure resistance	*p* < 0.01
CF1	Carvedilol	20	1.6	6 Hz (22 mA)	↑ seizure resistance	*p* < 0.001
				6 Hz (44 mA)	↑ seizure resistance	*p* < 0.01
				PTZ (85 mg/kg)	↑ latency to first GTCS	*p* < 0.01
CF1	Nilvadipine	1–10	0.1–0.8	6 Hz (22 mA)	No statistically significant difference	NS
CF1	Fenfluramine	1–30	0.1–2.4	6 Hz (22 mA)	10 and 17 mg/kg FF increased seizure resistance	*p* ≤ 0.05
RL/+	Amitriptyline	10	0.8	6 Hz (16 mA)	No statistically significant difference	NS
				PTZ (100 mg/kg)	No statistically significant difference	NS
RL/+	Carvedilol	20	1.6	6 Hz (16 mA)	↑ seizure resistance	*p* < 0.01
				PTZ (100 mg/kg)	↑ latency to first GTCS	*p* ≤ 0.05
RL/+	Fenfluramine	17	1.4	6 Hz (16 mA)	No statistically significant difference	NS

NS- not significant.

^a^ Human dose calculated based on Nair and Jacob (2016) and the average weight of an adult human ∼62 kg.

We observed a dose-dependent response with carvedilol, in which increasing CVD doses provided greater protection against 6 Hz-induced seizures, with maximum protection achieved with 20 mg/kg CVD (Figure 1E). At this dose of CVD, six of 8 CF1 mice were protected against 6 Hz seizures (RS0, Figure 1E). The ability of this dose to effectively block 6 Hz-induced seizures was confirmed in a separate cohort of CF1 mice (Figure 1F). Unlike AMI, CVD (20 mg/kg) also conferred significant protection when the CF1 mice were tested at 2xCC (Figure 1G). CVD was also able to significantly increase the latency to the first GTCS following PTZ administration, and 50% of the CVD-treated mice (5 of 10 mice) did not exhibit a GTCS during the 30-min observation period (Figure 1H).

In contrast to AMI and CVD, nilvadipine did not significantly protect against 6 Hz seizures at any of the doses tested (Figure 1I). We were unable to prepare higher doses of nilvadipine due to its low solubility; thus, this compound was not examined further. Finally, we also observed a dose-dependent increase in protection against 6 Hz seizures with fenfluramine, with doses of 10 and 17 mg/kg leading to increased resistance against 6 Hz seizures when tested in CF1 mice (Figure 1J).

### Carvedilol also provides robust protection against induced seizures in *Scn8a* RL/+ mutant mice

**FIGURE 2:**
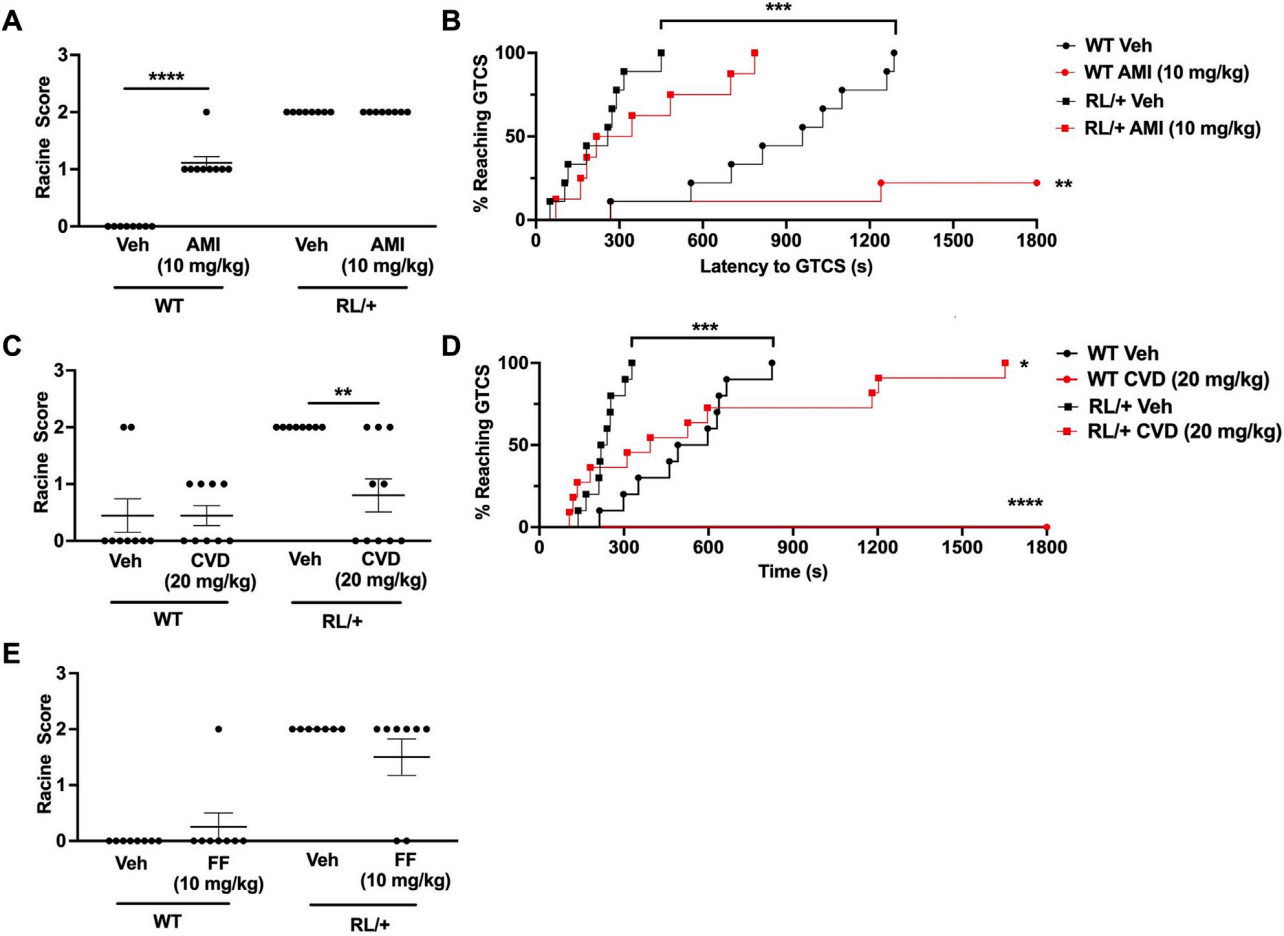
Carvedilol increases seizure resistance in RL/+ mutants. **(A)** Amitriptyline (AMI, 10 mg/kg) had no effect on seizure susceptibility in the RL/+ mutants, but slightly increased seizure susceptibility in the wild-type (WT) littermates. **(B)** AMI increased the latency to the first GTCS in the RL/+ mutants and WT littermates. Of the AMI-treated WT mice, seven of nine mice did not exhibit a GTCS during the 30-min observation period. **(C)** Carvedilol (20 mg/kg) was able to increase resistance against 6 Hz-induced seizures in the RL/+ mutants. **(D)** Carvedilol significantly increased the latency to the first GTCS following PTZ administration in the RL/+ mutants. All of the WT littermates treated with CVD were completely protected against PTZ-induced seizures. **(E)** Fenfluramine (17 mg/kg) had no effect on susceptibility to 6 Hz-induced seizures in the RL/+ mutants. *p ≤ 0.05, **p < 0.01, ***p < 0.001, ****p < 0.0001.

Since AMI, CVD, and FF conferred seizure protection in CF1 mice, we next investigated whether these compounds could similarly protect against induced seizures in the *Scn8a* RL/+ mutants. Surprisingly, we found that 30 mg/kg AMI caused excessive sedation and 5–10^o^C reduction in body temperature in the RL/+ mutants and their WT littermates, alterations that were not observed in the CF1 mice. We also observed myoclonic jerks in two RL/+ mutants that were administered 30 mg/kg AMI. Thus, we tested a lower AMI dose (10 mg/kg) in the RL/+ mutants and WT littermates in order to avoid these side effects. However, the lower AMI dose did not protect against 6 Hz seizures in the RL/+ mutants and appeared to slightly increase susceptibility to 6 Hz seizures in the WT littermates (Figure 2A). In contrast, AMI (10 mg/kg) did prevent PTZ-induced GTCSs during the 30-min observation period in 7 of 9 (78%) WT littermates. However, there was no statistically significant difference in the latency to the first GTCS or the number of mice exhibiting a GTCS between the AMI- and vehicle-treated RL/+ mutants following PTZ administration (Figure 2B).

Similar to its effect in CF1 mice, we found that CVD (20 mg/kg) was able to protect against induced seizures in the RL/+ mutants (Figures 2C, D). Unlike vehicle-treated RL/+ mutants which all exhibited RS2 seizures in the 6 Hz paradigm, 50% (5 of 10) of CVD-treated RL/+ mutants were completely protected (RS0), and 2 CVD-treated RL/+ mutants displayed a less severe response (RS1). CVD was also able to significantly increase the latency to the first PTZ-induced GTCS in the RL/+ mutants (Figure 2D). Furthermore, PTZ-induced GTCSs were not observed in any of the WT littermates that were treated with CVD during the 30-min observation period.

Finally, we examined the ability of fenfluramine to protect against 6 Hz seizures in the RL/+ mutants. In contrast to the CF1 mice, we found that fenfluramine (17 mg/kg) was only able to prevent a 6 Hz-induced seizure in 2 RL/+ mutants (RS0), while the remaining treated mutants seized (RS2; Figure 2E). At a higher fenfluramine dose (30 mg/kg), we observed proconvulsant effects in CF1 mice and significant side effects including hyperactivity and head twitching in the RL/+ mutants and WT littermates (not shown). Thus, we did not proceed with further testing of fenfluramine in the RL/+ mutants.

## Discussion

Using an *in vitro* drug screen, Atkin and others found that amitriptyline, carvedilol, and nilvadipine were able to reduce sodium influx and inhibit sodium currents (Atkin et al., 2018). Given that *SCN8A* mutations are often gain-of-function and result in increased sodium currents, it was speculated that these drugs might be efficacious in *SCN8A*-derived epilepsy. In addition, fenfluramine has garnered interest for its ability to significantly reduce seizure frequency in several severe pediatric forms of epilepsy. A recent study also reported a significant reduction in seizure frequency in three patients with *SCN8A* epilepsy mutations who were treated with fenfluramine (Aledo-Serrano et al., 2022).

To further explore the clinical potential of these compounds, we tested their ability to increase resistance to induced seizures in CF1 mice and a mouse model of *Scn8a-*derived epilepsy. We found that AMI and CVD provided robust protection against 6 Hz- and PTZ-induced seizures in CF1 WT mice; however, at the doses tested, nilvadipine did not alter seizure susceptibility (Figure 1). The dose range of nilvadipine that we tested was based on previous mouse studies (Morin et al., 2018; Morin et al., 2020); thus, it is possible that higher doses might be required to increase seizure resistance. However, due to the limited solubility of this compound, we were unable to test higher doses.

When administered to RL/+ mutants and WT littermates, we found that CVD but not AMI, was able to increase resistance against 6 Hz- and PTZ-induced seizures (Figures 2C,D). This observation was surprising given the evidence that both AMI and CVD can inhibit sodium channels (Barber et al., 1991; Bankston and Kass, 2010). Furthermore, AMI has high affinity for binding to the open and inactivated sodium channel (Wang et al., 2004), which is a feature of several drugs that have antiseizure effects (Xie et al., 1995). The electrophysiological assays conducted by Atkin et al., also suggested that AMI has a greater inhibitory effect on Na_v_1.6 channels compared to CVD, nilvadipine, and carbamazepine (Atkin et al., 2018). It is possible that other factors, such as the bioavailability or half-life of AMI, or the biophysical effect of the R1620L mutation, could have influenced the effect of AMI in the RL/+ mutants.

While a recent study reported that fenfluramine was able to significantly reduce seizure frequency in three patients with *SCN8A-*derived epilepsy (Aledo-Serrano et al., 2022), we did not observe any significant effect on susceptibility to 6 Hz-induced seizures in RL/+ mutants treated with fenfluramine (Figure 2E). We did not test higher doses of fenfluramine in the RL/+ mutants as a higher dose of fenfluramine (30 mg/kg) in CF1 WT mice was associated with increased seizure susceptibility and significant side effects were observed in the RL/+ mutants and WT littermates.

In the current study, we found that only CVD was able to increase resistance to 6 Hz- and PTZ-induced seizures in the RL/+ mutants. While there is no standard of care treatment for patients with *SCN8A*-derived epilepsy, from several published studies, sodium channel blockers like oxcarbazepine (OXC) can provide some seizure protection and are well-tolerated (Gardella et al., 2018; Talwar and Hammer, 2021; Johannesen et al., 2022). We previously observed a dose-dependent increase in OXC-mediated seizure protection in RL/+ mutants, with 50% and 100% of the mutants being protected against 6 Hz seizures with 15–20 mg/kg and 50 mg/kg OXC, respectively (Wong et al., 2021a). In the current study, approximately 50% of the RL/+ mutants were protected against 6 Hz seizures with 20 mg/kg CVD treatment.

Altogether, we found that the serotonergic drugs, AMI and fenfluramine, did not increase seizure resistance in the RL/+ mutants; however, CVD, which is a β-adrenergic receptor blocker, was able to increase seizure resistance. Previous studies have suggested a role for β-adrenergic receptors in modulating seizure susceptibility (Lints and Nyquist-Battie, 1985; Goel, 2013). Activation of β-adrenergic receptors contribute to the generation and propagation of audiogenic seizures (Lints and Nyquist-Battie, 1985), while antagonism of the β-adrenergic receptor increases seizure resistance (Goel, 2013). CVD has been shown to increase seizure resistance and potentiate the antiseizure effects of sodium valproate in WT mice (Goel, 2013). In addition, CVD treatment decreased the duration and severity of post-ischemic seizures in a rat model (El-Kharashi and Abd El Samad, 2011). However, Wengert et al., observed no protection against audiogenic seizure-induced mortality following administration of the β-adrenergic receptor blocker sotalol in adult mice expressing the *SCN8A* N1768D mutation (Wengert et al., 2021). Thus, further research will be required to better understand the therapeutic potential of modulating the noradrenergic system in *SCN8A*-derived epilepsy.

Common biophysical effects of *SCN8A* epilepsy mutations include greater levels of persistent and/or resurgent sodium currents (Lopez-Santiago et al., 2017; Wengert et al., 2019; Pan and Cummins, 2020; Tidball et al., 2020). These underlying biophysical abnormalities provide the opportunity for targeted treatment development which might result in greater efficacy and less side effects when compared to other drugs. In the current study, we tested AMI, CVD, and nilvadipine in the *Scn8a* RL/+ mutants as these drugs demonstrated the ability to inhibit sodium current influx *in vitro*. We also examined fenfluramine based on recent clinical evidence suggesting that it might be efficacious in *SCN8A* *-*derived epilepsy. Other drugs that target persistent and/or resurgent sodium currents have recently been described. For example, Prax330 was shown to reduce persistent and resurgent sodium currents in subiculum neurons isolated from *Scn8a* N1768D/+ mutant mice (Wengert et al., 2019). Cenobamate (YKP3089), which received FDA approval for the treatment of focal seizures in adults in 2019, works, in part, by enhancing the inactivated state of VGSCs and inhibiting persistent sodium currents (Nakamura et al., 2019). Based on its mechanism of action, we speculate that cenobamate might also be beneficial in *SCN8A-*associated epilepsy. Finally, Johnson et al. recently reported the development of a Na_v_1.6 selective sodium channel inhibitor (NBI-921352) that blocks persistent and resurgent sodium currents (Johnson et al., 2022). NBI-921352 was able to inhibit firing of glutamatergic neurons and increase seizure resistance in *Scn8a* N1768D/+ mutant mice (Johnson et al., 2022). Whether these observations in preclinical rodent models translate to humans has not yet been established. While it is beneficial to develop novel drugs for *SCN8A-*derived epilepsy, there is also value in testing FDA-approved compounds, such as CVD, as repurposing drugs could provide faster clinical application.

In summary, we evaluated the ability of four drugs to increase resistance to induced seizures in a mouse model of *SCN8A* epilepsy. Considering the differences in drug metabolism between mice and humans (Nair and Jacob, 2016), the doses of the drugs we tested fell within the range of doses administered in humans (Table 1). AMI, CVD, and nilvadipine are currently used in the treatment of conditions such as heart disease and depression; thus, the doses we tested in the mice may not correspond to a seizure-protective dose in humans. Fenfluramine is currently used as an add-on therapy in several forms of pediatric epilepsy, and while the doses we tested are within the current range used in patients with epilepsy (0.2–0.7 mg/kg/day), we only examined the effect of fenfluramine as a monotherapy treatment in the mice. It is possible we may observe greater seizure protection when using fenfluramine as an add-on treatment. Overall, we found that carvedilol, a β-adrenergic receptor blocker, robustly increased resistance to 6 Hz- and PTZ-induced seizures in the RL/+ mutants. While this mouse line offered the advantage of a *SCN8A* mutation with both gain-of-function and loss-of-function properties, RL/+ mutants exhibit infrequent spontaneous seizures, and thus, we were unable to evaluate whether carvedilol could also reduce spontaneous seizure frequency. In future studies, it would be important to also evaluate the effect of these drugs in female RL/+ mutants and WT littermates. The results of this study and previous evidence for the role of noradrenergic modulation in *SCN8A*-associated epilepsy, provide support for the continued investigation of the potential of CVD and other β-adrenergic receptor blockers as treatments for *SCN8A-*associated epilepsy.

## Data Availability

The raw data supporting the conclusion of this article will be made available by the authors, without undue reservation.
